# Influence of vaccination against infectious diseases on the carbon footprint of fattening pigs: a systematic review

**DOI:** 10.3389/fvets.2024.1487742

**Published:** 2024-12-18

**Authors:** Julia Gickel, Clara Berenike Hartung, Amr Abd El-Wahab, Julia Hankel, Christian Visscher

**Affiliations:** ^1^Science and Innovation for Sustainable Poultry Production (WING), University of Veterinary Medicine Hannover, Foundation, Bakum, Germany; ^2^Institute for Animal Nutrition, University of Veterinary Medicine Hannover, Foundation, Hanover, Germany

**Keywords:** carbon footprint, fattening pigs, performance, LCA, vaccination, porcine circovirus type 2, *Mesomycoplasma hyopneumoniae*, *Lawsonia intracellularis*

## Abstract

**Introduction:**

In all sectors of the economy, including livestock production, there is an increasing focus on sustainability criteria. The carbon footprint is therefore an important target value in pig production. The aim is to minimize this value. Infectious diseases may affect the performance negatively, potentially leading to a higher carbon footprint. Therefore, vaccinations may be a useful tool to ensure a high level of sustainability in pork production.

**Materials and methods:**

The aim of this evaluation was to assess the impact of vaccinations against Porcine Circovirus Type 2 (PCV2), *Mesomycoplasma hyopneumoniae* (*M. hyo*), both PCV2 and *M. hyo*, and *Lawsonia intracellularis* (LI) in epidemic situations in Europe on performance parameters using results from former publications on these diseases. These parameters were then used to calculate the carbon footprint of the pigs using life cycle assessment (LCA). The data collection with PubMed was based on the PRISMA guidelines for systematic reviews and meta-analyses, from which, however, some deviations were made. In total, 14 trials on PCV2, 10 trials on *M. hyo*, 14 trials on *M. hyo* and PCV2, and 17 trials on LI fulfilled the criteria and were included in this evaluation. In general, review articles and studies published before 1990 were excluded as were studies with incomplete data concerning the performance parameters and studies from non-European countries if the published body weights, genetics used, or other details in the experimental setup suggested they were not comparable to European standards.

**Results:**

The mean carbon footprint was up to 12.1% (PCV2), 2.5% (*M. hyo*), 9.3% (PCV2 and *M. hyo*), or 3.5% (LI) lower following a vaccination.

**Discussion:**

This evaluation clearly shows that healthy animals can achieve a reduced carbon footprint through better performance with lower resource consumption, which is extremely important for sustainable animal husbandry. The use of health preserving measures such as vaccination can be a useful and important tool for reaching this goal.

## 1 Introduction

In every sector of the economy, including animal husbandry, there is a growing emphasis on sustainability criteria, referred to as ESG (environmental, social, and governance) criteria (1). The impact of livestock farming systems can be assessed through life cycle assessments (LCA). This method, which originated in industry during the 1960's, was adapted for agriculture in the 2000's (2). It is globally recognized and standardized by International Organization for Standardization (ISO) 14040 and ISO 14044, which were recently updated in 2020 (3). The European Commission has also released a Product Environmental Footprint (PEF) guide to standardize LCA studies across Europe (4). Beyond this general guidance, Product Environmental Footprint Category Rules (PEFCR) can be established to enhance the reproducibility of specific LCAs (4). In the livestock sector, the Livestock Environmental Assessment and Performance Partnership (LEAP) offers additional guidance, and the Global Feed LCA Institute (GFLI) has created a methodology aligned with PEF and LEAP specifically for animal feed (5, 6).

Infectious diseases in fattening pigs are known to cause symptoms like diarrhea, reduced weight gain and feed intake, and in severe cases, increased mortality (7–14). Porcine circovirus type 2 (PCV2), *Mesomycoplasma hyopneumoniae* (*M. hyo*), formerly called *Mycoplasma hyopneumoniae*, and *Lawsonia intracellularis* (*LI*) are important pathogens in pigs leading to substantial economic impacts (15–18). For all of them, vaccination is an important tool to control infections and symptoms of an infection and is used worldwide (16–19).

Infections with the PCV2 were firstly described in Canada in the 1990's (20, 21) as postweaning multisystemic wasting syndrome (PMWS), a disease with symptoms like wasting, respiratory problems, paleness of the skin or icterus, and sometimes diarrhea in weaning piglets and fattening pigs (9). Later, PCV2 infections were reported worldwide with a great variety of clinical features such as PCV2-systemic disease (PCV2-SD), Porcine dermatitis and nephropathy syndrome (PDNS), and PCV2-reproductive disease (stillbirths and mummifications), which are summarized as Porcine circovirus diseases (PCVD) and can occur in piglets as well as fattening and breeding sows (10). The former described PCV1 was identified as non-pathogenic in pigs (22). PCV3, firstly identified in 2016, however, can cause symptoms similar to PCV2, including porcine dermatitis and nephropathy syndrome (PDNS) and reproductive disorders (23, 24). In 2019, a new circovirus was reported from several pigs in China, designated as PCV4, causing PDNS-like symptoms and severe respiratory and enteric symptoms (25, 26). Estimations on both novel porcine circoviruses, PCV3 and PCV4, show that they might have the potential to contribute to high economic losses similar to PCV2, but long-term data are missing due to a limited period of awareness (25, 27, 28).

*M. hyo* is the main agent causing the chronic respiratory enzootic pneumonia (EP) in pigs. It is also essentially involved in the porcine respiratory disease complex (8, 12, 17). Infections with *M. hyo* occur worldwide, with a huge economic impact to the pig industry mainly due to impaired performance and enhanced mortality caused by secondary infections as well as costs for therapy and vaccination (17, 29). Infections with *M. hyo* occur mainly in fattening pigs, but also weaning piglets and breeding sows can be affected (30, 31).

Infections with *LI* are present worldwide and cause major economic losses due to reduced daily weight gain, poor feed conversion ratio, and increased mortality (14, 32, 33). The disease is characterized by thickening of the intestinal epithelium of the ileum and proximal colon due to enterocyte proliferation (13) and could be found in two clinical presentations in all age and production categories, mostly in fattening pigs (33). The chronic proliferative form is known as proliferative enteropathy (PE) with relatively low mortality (34), and the acute infection is known as proliferative hemorrhagic enteritis (PHE), which can cause sudden death (14, 35).

The carbon footprint of fattening pigs is closely linked to the performance of the animal. Infectious diseases may affect the performance negatively, potentially leading to a reduction in sustainability. Therefore, vaccinations may be a useful tool to ensure a high level of sustainability in pork production. This evaluation aimed to examine the impact of vaccinations against PCV2, *M. hyo*, both PCV2 and *M. hyo*, and *LI* in epidemic situations on the carbon footprint of fattening pigs.

## 2 Materials and methods

Based on a systematic literature review using the database PubMed, the effects of vaccinations against PCV2, *M. hyo*, both PCV2 and *M. hyo*, and *LI* in epidemic situations on the performance of fattening pigs, primarily in Europe, were monitored. Data on body weight development, feed conversion ratio (FCR), and mortality were extracted from published studies. In addition to scientific journal articles, papers held at international scientific conferences were consulted to enhance the data set. The data collection and further presentation were based on the PRISMA guidelines for systematic reviews and meta-analyses (36), from which, however, some deviations were made as this evaluation does not assess the risk of bias across studies. However, this was examined in more detail in the discussion. Review articles were excluded from the data collection process, since they did not publish original data and were generally selected and summarized according to specific criteria that might not align with this evaluation. Studies published before 1990 were also excluded due to significant differences in husbandry standards compared to the present day. Additionally, studies from non-European countries were excluded if the published body weights, genetics used, or other details in the experimental setup suggested they were not comparable to European standards. The flow charts according to PRISMA for each vaccination may be found in the Supplementary material.

Data were extracted from trials in the rearing period, trials in the fattening period, and trials including the rearing and the fattening period. For later distinction each trial was assigned to one of these categories exclusively according to their age. In total, 14 trials on PCV2, 10 trials on *M. hyo*, 14 trials on *M. hyo* and PCV2, and 17 trials on *LI* fulfilled the criteria and were included in this evaluation (Table 1).

**Table 1:** Overview of number of trials included per category concerning porcine circovirus type 2 (PCV2), *Mesomycoplasma hyopneumoniae* (*M. hyo)*, PCV2 and *M. hyo*, and *Lawsonia intracellularis* (*LI)*.

	**PCV2**	***M. hyo***	**PCV2 and *M. hyo***	***LI***
Total number of included trials (time of publication)	14	10	14	17
	(2007–2020)	(1999–2017)	(2012–2022)	(2004–2022)
Number of included trials concerning the rearing period (time of publication)	2	No data	2	No data
	(2016–2017)		(2017–2018)	
Number of included trials concerning the fattening period (time of publication)	4	3	7	15
	(2012–2020)	(1999–2017)	(2012–2020)	(2012–2020)
Number of included trials concerning the rearing period and the fattening period (time of publication)	6	6	5	2
	(2007–2018)	(2012–2017)	(2012–2020)	(2018–2022)
References^a, b^	Astrup, 2017	Arsenakis, 2017	Agerley, 2012	Antonelli, 2021
	Brons, 2010	Beek, 2017	Beek, 2017	Gómez, 2010
	Coll, 2012	Beffort, 2017	Boulbria, 2021	Kolb, 2004
	de Groot, 2014	Herrera, 2014	Cho, 2022	Marcos, 2022
	Fachinger, 2007	Kristensen, 2014	Duivon, 2016	Meschede, 2021
	Fiebig, 2007	Maes, 1999	Ju, 2012	Musse, 2023a
	Jansen, 2020	Struik, 2014	Nielsen, 2018	Musse, 2023b
	Kaalberg, 2017	Tassis, 2012	Pagot, 2017	Nieberding, 2022
	Koenders, 2012	Tzika, 2015	Pelz, 2020	Peiponen, 2018
	Lewandowski, 2012		Tassis, 2017	Raymakers and Kraneburg, 2016
	Nielsen, 2017		Van Hee, 2016	
	Rahm, 2018			
	Yao, 2010			

^a^One publication may include more than one trial.

^b^These references may be found in the Supplementary material.

Data collection included the start, end, and duration of the trial (in days). The initial body weight (kg), final body weight (kg), average daily gain (ADG, g per day), FCR (kg feed per kg gain), and mortality (%) of the fattening pigs were extracted for each group (epidemically infected/non-vaccinated control and epidemically infected/vaccinated trial group). The mean values of trial duration, ADG, FCR, and mortality were then used to perform an LCA to determine the carbon footprint (CO_2_ eq per kg weight gain) of this specific phase for each group. For different trial groups (e.g., different times of vaccination), the mean value of both trial groups was used. If data on body weight development were incomplete, standard values from the information on fattening pigs derived from the Bavarian State Research Center for Agriculture (LfL) were used to estimate the initial body weight (37), and a missing final body weight was calculated using the initial body weight, ADG, and trial duration. In the absence of mortality data, a value of 1.7% was assumed for trials within the rearing period, and 2.6% was assumed for trials within the fattening period (38).

The calculation of the carbon footprint was conducted using the Opteinics^TM^ software application (version 2.2), developed based on ISO 14040 and ISO 14044 standards. Only the respective test phase was considered in each case; the production of piglets and the slaughtering process were not included, as their effects can be regarded as nearly identical for both non-vaccinated and vaccinated fattening pigs. The calculated value only accounted for additions during the trial period, described as CO_2_ eq per kg body weight gain. Due to the mentioned assumptions and exclusions, the values provided were not representative of fattening pigs in general.

To ensure the highest possible comparability, a feed with an identical composition was assumed for all calculations. A one-phase piglet feed (up to week 11 of life) and a three-phase feeding system in the fattening period were used. All rations were formulated based on wheat (Germany), barley (Germany), and soybean meal (Brazil). Details on all rations may be found in the Supplementary material. Results from other regions with a lower carbon footprint for soy extraction meal (like North America or possibly Europe) were not included in this review, resulting in comparatively high absolute values for the feed, as deforestation effects were accounted for. The values used by the software for the carbon footprint of the raw materials were provided by the GFLI database, which was created based on the PEF and LEAP guidelines.

For water consumption, the reference value of 8.67 liters per day, provided by the Opteinics^TM^ software application, was used. This includes drinking water and water for cooling and cleaning. This value represents an average across the entire production phase and was not adjusted for studies depicting early or late trial periods, as water consumption contributes only marginally to the carbon footprint. Fuel and energy consumption, included in general LCA studies for pigs, were assumed to be zero for all calculations to clearly attribute the results to disease effects without distortion from varying trial lengths or differing energy requirements of younger and older animals.

To estimate the annual savings potential of the carbon footprint through the vaccination against infectious diseases in epidemically infected situations, a scenario calculation was carried out exemplary for Germany comparing vaccinated to non-vaccinated animals. It was assumed that the carbon footprint of all pigs slaughtered in Germany was 3.6 kg CO_2_ eq per kg weight gain. Additionally, it was assumed, based on the literature, that 43.8 million pigs were slaughtered per year (39) with a mean carcass yield of 79% and a mean carcass weight of 97.2 kg (40).

## 3 Results

### 3.1 Effects on the carbon footprint of a vaccination against porcine circovirus type 2

#### 3.1.1 Consideration of all publications

In total, data from 14 trials, published from 2007 to 2020, fulfilled the criteria and were included in this evaluation regarding a vaccination against PCV2 in epidemically infected situations (Table 1). The trials started between day 16 and 75 of life and ended between day 42 and 194 of life, including a trial duration of 21–168 days. On average, the trials started at day 42 of life and ended at day 166 of life; the mean trial duration was 125 days (Table 2).

**Table 2:** Means ± standard deviations of parameters (age of animals at start and end of trial, duration of trial, body weight of animals at start and end of trial, ADG, feed conversion ratio, and mortality) in non-vaccinated and vaccinated groups of considered trials concerning an infection with porcine circovirus type 2.

**Parameter^a^**	**Mean of all trials** ^**b**^		**Mean of trials for rearing period** ^**b**^		**Mean of trials for fattening period** ^**b**^		**Mean of trials for rearing and fattening period** ^**b**^	
Age at start of trial [days]	42 ± 24 (*n* = 12)		25 ± 4 (*n* = 2)		75 ± 0 (*n* = 4)		26 ± 5 (*n* = 6)	
Age at end of trial [days]	166 ± 51 (*n* = 12)		58 ± 16 (*n* = 2)		194 ± 0 (*n* = 4)		183 ± 22 (*n* = 6)	
Duration of trial [days]	125 ± 46 (*n* = 12)		34 ± 13 (*n* = 2)		120 ± 0 (*n* = 4)		158 ± 18 (*n* = 6)	
Non-vaccinated (nv) or vaccinated groups (vac)	nv	vac	nv	vac	nv	vac	nv	vac
Body weight at start of trial [kg]	14.4 ± 9.8	14.4 ± 9.8	8.0 ± 0.0	8.0 ± 0.0	28.2 ± 0.4	28.2 ± 0.4	7.3 ± 1.0	7.4 ± 0.9
	(*n* = 12)	(*n* = 12)	(*n* = 2)	(*n* = 2)	(*n* = 4)	(*n* = 4)	(*n* = 6)	(*n* = 6)
Body weight at end of trial [kg]	99.8 ± 36.5	103.4 ± 38.5	20.8 ± 8.3	20.7 ± 8.1	118.7 ± 4.6	122.5 ± 6.9	113.5 ± 11.0	118.2 ± 13.0
	(*n* = 12)	(*n* = 12)	(*n* = 2)	(*n* = 2)	(*n* = 4)	(*n* = 4)	(*n* = 6)	(*n* = 6)
Average daily gain [g per day]	663 ± 217	692 ± 222	336 ± 122	337 ± 118	823 ± 81	855 ± 66	608 ± 127	640 ± 121
	(*n* = 13)	(*n* = 13)	(*n* = 2)	(*n* = 2)	(*n* = 4)	(*n* = 4)	(*n* = 6)	(*n* = 6)
Feed conversion ratio [kg per kg]	2.58 ± 0.23	2.46 ± 0.24	2.01	1.89	2.67 ± 0.15	2.59 ± 0.13	2.59 ± 0.05	2.43 ± 0.12
	(*n* = 9)	(*n* = 9)	(*n* = 1)	(*n* = 1)	(*n* = 4)	(*n* = 4)	(*n* = 3)	(*n* = 3)
Mortality rate [%]	6.9 ± 8.3	3.5 ± 2.0	3.1	2.4	2.3 ± 0.6	2.0 ± 0.5	12.4 ± 10.0	5.3 ± 1.7
	(*n* = 10)	(*n* = 10)	(*n* = 1)	(*n* = 1)	(*n* = 4)	(*n* = 4)	(*n* = 4)	(*n* = 4)

^a^All values of a category are calculated directly from the raw data. Therefore, the values cannot be calculated separately;

^b^n represents the number of considered trials.

The ADG of non-vaccinated animals in epidemically infected situations ranged from 214 to 1,014 g, the mean was 663 ± 217 g. In the epidemically infected groups that also received a vaccination, the ADG ranged between 219 and 1,060 g, while the mean was 692 ± 222 g, this corresponding to a mean increase of 4.3%. The feed conversion ratio of the animals in the non-vaccinated groups showed values between 2.01 and 2.85; the mean value was 2.58 ± 0.23. In comparison, the vaccinated groups with epidemic infection showed values between 1.89 and 2.75 with a mean value of 2.46 ± 0.24, this corresponding to a reduction of 4.7% (mean value). The mortality rate of animals in the non-vaccinated groups ranged from 1.3 to 29.6%; the mean was 6.9 ± 8.3%. In the vaccinated groups, the mortality rate ranged from 1.1 to 7.0%, while the mean value was 3.5 ± 2.0%, this corresponding to a reduction of 49.3%.

The calculation of the mean values of the carbon footprint resulted in a value of 3.56 kg CO_2_ eq per kg weight gain in the groups with an epidemic infection without vaccination and in a mean value of 3.31 kg CO_2_ eq per kg weight gain in the vaccinated groups with an epidemic infection, the latter corresponding to a reduction of 7.0%.

#### 3.1.2 Publications concerning the rearing period

Data from two of the 14 trials could be included for consideration concerning the rearing period. These were published in 2016 and 2017. The trials started at day 21 or 29 of life (mean: 25 days) and lasted 21 and 46 days (mean: 34 days), resulting in the age of 42 and 74 days of life (mean: 58 days) at the end of the trial (Table 2).

The ADG of the non-vaccinated groups was 214 g and 457 g, resulting in a mean of 336 ± 122 g. In the epidemically infected groups that also received a vaccination, the ADG was 219 and 454 g, resulting in a mean of 337 ± 118 g, this corresponding to a mean increase of 0.3%. The feed conversion ratio of the pigs in the non-vaccinated group (*n* = 1) showed a value of 2.01. In comparison, the corresponding vaccinated group showed a value of 1.89, this corresponding to a reduction of 6.0%. The mortality in the group without vaccination (*n* = 1) amounted to 3.1%. In the vaccinated group, the mortality rate was 2.4%, this corresponding to a reduction of 22.6%.

The calculation of the mean values of the carbon footprint resulted in a value of 1.43 kg CO_2_ eq per kg weight gain for the respective trial period in piglet rearing in the non-vaccinated groups and a value of 1.36 kg CO_2_ eq per kg of piglet gain in the vaccinated groups, the latter corresponding to a reduction of 4.9%.

#### 3.1.3 Publications concerning the fattening period

Data from four of all 14 trials could be included for consideration regarding the fattening period. These were published between 2012 and 2020. All of these trials started at day 75 of life and ended at day 194 of life, with a trial duration of 120 days (Table 2).

The ADG of the animals in the non-vaccinated groups during the fattening period ranged from 761 g to 961 g with a mean of 823 ± 81 g. In the vaccinated groups, the ADG values ranged between 792 g and 962 g, the mean value was 855 ± 66 g, this corresponding to an increase of 3.9%. The feed conversion ratio of the animals in the non-vaccinated groups showed values between 2.47 and 2.85; the mean value was 2.67 ± 0.15. In comparison, the feed conversion in the vaccinated groups ranged from 2.43 to 2.75; the mean was 2.59 ± 0.13, this corresponding to a reduction of 3.0%. The mortality rate of animals in the non-vaccinated groups ranged from 1.3 to 3.1%; the mean was 2.3 ± 0.6%. In the vaccinated groups, the mortality rate ranged between 1.1 and 2.5%, while the mean was 2.0 ± 0.5%, this corresponding to a reduction of 13.0%.

The calculation of the mean values of the carbon footprint resulted in a value of 3.38 kg CO_2_ eq per kg weight gain in the non-vaccinated groups and a value of 3.29 kg CO_2_ eq per kg weight gain in the vaccinated groups for the respective trial period in the fattening period, the latter corresponding to a reduction of 2.7%.

#### 3.1.4 Publications concerning the rearing and fattening period

Data from six of the 14 trials could be included in the consideration concerning the rearing and fattening period. These were published between 2007 and 2018. The trials started between day 16 and 29 of life and ended between day 133 and 194 of life, with a trial duration of 117–168 days. On average, the trials started at day 26 of life and ended at day 183 of life; the mean trial duration was 158 days (Table 2).

The ADG of the non-vaccinated animals in the rearing and fattening period ranged from 328 to 692 g; the mean value was 608 ± 127 g. In the vaccinated groups, the ADG ranged from 377 to 729 g, while the mean value was 640 ± 121 g, this corresponding to an increase of 5.3%. The feed conversion ratio of the animals in the non-vaccinated groups showed values between 2.53 and 2.65; the mean value was 2.59 ± 0.05. In comparison, the feed conversion ratio ranged from 2.28 to 2.56 in the vaccinated groups; the mean was 2.43 ± 0.12, this corresponding to a reduction of 6.2%. The mortality rate of the animals in the non-vaccinated groups was between 5.1 and 29.6%; the mean value was 12.4 ± 10.0%. In the vaccinated groups, the mortality rate ranged between 2.8 and 7.0%, while the mean value was 5.3 ± 1.7%, this corresponding to a reduction of 57.3%.

The calculation of the mean values of the carbon footprint resulted in a value of 3.88 kg CO_2_ eq per kg weight gain for the respective trial period in the non-vaccinated groups and a value of 3.41 kg CO_2_ eq per kg weight gain for that in the vaccinated groups, the latter corresponding to a reduction of 12.1%.

### 3.2 Effects on the carbon footprint of a vaccination against *Mycoplama hyopneumoniae*

#### 3.2.1 Consideration of all publications

In total, data from 10 trials, published from 1999 to 2017, fulfilled the criteria and were included in this evaluation regarding a vaccination against *M. hyo* in epidemically infected situations (Table 1). The trials started between day 7 and 75 of life and ended between day 165 and 206 of life, with a trial duration of 120–188 days. On average, the trials started at day 34 of life and ended at day 188 of life; the mean trial duration was 155 days (Table 3).

**Table 3:** Means ± standard deviations of parameters (age of animals at start and end of trial, duration of trial, body weight of animals at start and end of trial, ADG, feed conversion ratio, and mortality rate) in non-vaccinated and vaccinated groups of considered trials concerning an infection with *Mesomycoplasma hyopneumoniae* (ND, no data).

**Parameter^a^**	**Mean of all trials** ^**b**^		**Mean of trials for rearing period** ^**b**^		**Mean of trials for fattening period** ^**b**^		**Mean of trials for rearing and fattening period** ^**b**^	
Age at start of trial [days]	34 ± 23 (*n* = 9)		ND		73 ± 3 (*n* = 3)		21 ± 8 (*n* = 6)	
Age at end of trial [days]	188 ± 22 (*n* = 9)		ND		198 ± 6 (*n* = 3)		184 ± 13 (*n* = 6)	
Duration of trial [days]	155 ± 22 (*n* = 9)		ND		126 ± 8 (*n* = 3)		164 ± 17 (*n* = 6)	
Non-vaccinated (nv) or vaccinated groups (vac)	nv	vac	nv	vac	nv	vac	nv	vac
Body weight at start of trial [kg]	10.4 ± 8.8	10.3 ± 8.7	ND	ND	26.1 ± 2.6	26.0 ± 2.9	5.5 ± 1.7	5.7 ± 1.6
	(*n* = 9)	(*n* = 9)			(*n* = 3)	(*n* = 3)	(*n* = 6)	(*n* = 6)
Body weight at end of trial [kg]	98.5 ± 14.2	100.3 ± 14.6	ND	ND	114.0 ± 4.2	116.1 ± 4.8	93.5 ± 12.7	95.3 ± 13.2
	(*n* = 9)	(*n* = 9)			(*n* = 3)	(*n* = 3)	(*n* = 6)	(*n* = 6)
Average daily gain [g per day]	670 ± 72	687 ± 68	ND	ND	702 ± 55	723 ± 54	663 ± 73	678 ± 67
	(*n* = 9)	(*n* = 9)			(*n* = 3)	(*n* = 3)	(*n* = 6)	(*n* = 6)
Feed conversion ratio [kg per kg]	2.85 ± 0.05	2.77 ± 0.06	ND	ND	2.79 ± 0.09	2.72 ± 0.08	ND	ND
	(*n* = 3)	(*n* = 3)			(*n* = 3)	(*n* = 3)		
Mortality rate [%]	3.2 ± 0.6	2.9 ± 0.7	ND	ND	2.9 ± 0.9	2.4 ± 0.9	3.2 ± 0.4	2.9 ± 0.6
	(*n* = 6)	(*n* = 6)			(*n* = 3)	(*n* = 3)	(*n* = 3)	(*n* = 3)

^a^All values of a category are calculated directly from the raw data. Therefore, the values cannot be calculated separately.

^b^n represents the number of considered trials.

The ADG of animals in the non-vaccinated groups was 585–766 g; the mean was 670 ± 72 g. In the groups that were also vaccinated against *M. hyo*, the ADG ranged from 605 to 775 g; the median was 687 ± 68 g. This corresponds to an increase of 2.5%. The feed conversion ratio of the pigs in the non-vaccinated groups showed values between 2.80 and 2.90; the mean was 2.85 ± 0.05. In comparison, the feed conversion ratio of animals in the vaccinated groups showed values ranging from 2.71 to 2.83, while the mean was 2.77 ± 0.06, this corresponding to a reduction of 2.8%. The mortality rate of non-vaccinated animals was between 2.6 and 4.0%; the mean value was 3.2 ± 0.6%. In the vaccinated groups, the mortality rate ranged from 1.9 to 3.8%, while the mean value was 2.9 ± 0.7%, this corresponding to a reduction of 9.4%.

The calculation of the mean values of the carbon footprint resulted in a value of 3.79 kg CO2 eq per kg weight gain in the non-vaccinated groups and a value of 3.69 kg CO2 eq per kg weight gaining the vaccinated groups, the latter corresponding to a reduction of 2.6%.

#### 3.2.2 Publications concerning the rearing period

None of the considered studies relating to *M. hyo* were exclusively concerned with the rearing period. Therefore, a corresponding analysis was not possible.

#### 3.2.3 Publications concerning the fattening period

Data from three of the 10 trials, published between 1999 and 2017, could be included in the consideration concerning the fattening period. The trials started between day 69 and 75 of life and ended between day 194 and 206 of life, with a trial duration of 120–138 days. On average, the trials started at day 73 of life and ended at day 198 of life; the mean trial duration was 126 days (Table 3).

The ADG of animals in the non-vaccinated groups in the fattening period ranged from 626 to 755 g; the mean was 702 ± 55 g. In the vaccinated groups, the ADG ranged between 648 and 775 g, while the mean value was 723 ± 54 g, this corresponding to an increase of 3.0%. The feed conversion rate of the animals in the non-vaccinated groups showed values between 2.68 and 2.90; the mean value was 2.79 ± 0.09. In comparison, the feed conversion rate of the animals in the vaccinated group ranged from 2.63 to 2.83, while the mean value was 2.72 ± 0.08, this corresponding to a reduction of 2.5%. The mortality rate of animals in the non-vaccinated groups was 2.0 to 4.0%; the mean value was 2.9 ± 0.9%. In the vaccinated groups, the mortality rate ranged from 1.7 to 3.8%, while the mean value was 2.4 ± 0.9%, this corresponding to a reduction of 17.2%.

The calculation of the mean values of the carbon footprint resulted in a value of 3.54 kg CO_2_ eq per kg weight gain for the respective trial period in the non-vaccinated groups and a value of 3.45 kg CO_2_ eq per kg weight gain in the vaccinated groups, the latter corresponding to a reduction of 2.5%.

#### 3.2.4 Publications concerning the rearing and fattening period

Data from seven of the 10 trials could be included in the consideration concerning the rearing and fattening period. These were published between 2012 and 2017. The trials started between day 16 and 29 of life and ended between day 133 and 194 of life, with a trial duration of 117–168 days. On average, the trials started at day 26 days of life and ended at day 183 of life; the mean trial duration was 158 days (Table 3).

The ADG of the non-vaccinated animals in the rearing and fattening period was 585–766 g; the mean was 663 ± 73 g. In the vaccinated groups, the ADG ranged between 605 and 766 g, while the mean value was 678 ± 67 g, this corresponding to an increase of 2.3%. The mortality rate of the non-vaccinated animals ranged between 2.7 and 3.7%; the mean value was 3.2 ± 0.4%. In the vaccinated groups, the mortality rate ranged between 2.1 and 3.6%, while the mean value was 2.9 ± 0.6%, this corresponding to a reduction of 9.4%.

A calculation of the carbon footprint concerning the average values for these publications was not possible due to missing data on the feed conversion ratio.

### 3.3 Effects on the carbon footprint of a vaccination against porcine circovirus type 2 and *Mycoplama hyopneumoniae*

#### 3.3.1 Consideration of all publications

In total, data from 14 trials, published from 2012 to 2022, fulfilled the criteria and were included in this evaluation regarding a vaccination against PCV2 and *M. hyo* in epidemically infected situations (Table 1). The trials started between day 21 and 75 of life and ended between days 74 and 194 of life, with a trial duration of 46–166 days. On average, the trials started at day 47 of life and ended at day 156 of life; the mean trial duration was 110 days (Table 4).

**Table 4:** Means ± standard deviations of parameters (age of animals at start and end of trial, duration of trial, weight of animals at start and end of trial, ADG, feed conversion ratio, and mortality rate) in non-vaccinated and vaccinated groups of considered trials concerning an infection with porcine circovirus type 2 and *Mesomycoplasma hyopneumoniae* (ND, no data).

**Parameter^a^**	**Mean of all trials** ^**b**^		**Mean of trials for rearing period** ^**b**^		**Mean of trials for fattening period** ^**b**^		**Mean of trials for rearing and fattening period** ^**b**^	
Age at start of trial [days]	47 ± 23 (*n* = 14)		29 ± 1 (*n* = 2)		70 ± 3 (*n* = 7)		22 ± 3 (*n* = 5)	
Age at end of trial [days]	156 ± 38 (*n* = 14)		76 ± 2 (*n* = 2)		165 ± 25 (*n* = 6)		176 ± 9 (*n* = 5)	
Duration of trial [days]	110 ± 40 (*n* = 14)		48 ± 2 (*n* = 2)		96 ± 22 (*n* = 6)		154 ± 8 (*n* = 5)	
Non-vaccinated (nv) or vaccinated groups (vac)	nv	vac	nv	vac	nv	vac	nv	vac
Body weight at start of trial [kg]	12.1 ± 9.2	12.0 ± 9.3	7.4 ± 0.6	7.4 ± 0.6	28.0 ± 0.0	28.0 ± 0.0	6.5 ± 1.0	6.4 ± 1.1
	(*n* = 13)	(*n* = 13)	(*n* = 2)	(*n* = 2)	(*n* = 2)	(*n* = 2)	(*n* = 4)	(*n* = 4)
Body weight at end of trial [kg]	92.0 ± 38.2	95.8 ± 41.9	29.0 ± 0.6	28.9 ± 0.2	137.7 ± 6.7	149.7 ± 11.0	99.0 ± 11.6	101.1 ± 11.0
	(*n* = 13)	(*n* = 13)	(*n* = 2)	(*n* = 2)	(*n* = 2)	(*n* = 2)	(*n* = 4)	(*n* = 4)
Average daily gain [g per day]	674 ± 163	700 ± 201	441 ± 3	439 ± 10	914 ± 56	1,014 ± 92	671 ± 44	678 ± 45
	(*n* = 9)	(*n* = 9)	(*n* = 2)	(*n* = 2)	(*n* = 2)	(*n* = 2)	(*n* = 4)	(*n* = 4)
Feed conversion ratio [kg per kg]	2.70 ± 0.59	2.58 ± 0.40	1.87	1.81	2.87 ± 0.5	2.63 ± 0.22	ND	ND
	(*n* = 7)	(*n* = 7)	(*n* = 1)	(*n* = 1)	(*n* = 5)	(*n* = 5)		
Mortality rate [%]	4.3 ± 2.0	3.3 ± 1.1	2.8 ± 0.4	3.8 ± 1.1	5.3 ± 2.2	2.5 ± 0.1	4.6 ± 1.9	3.4 ± 1.1
	(*n* = 9)	(*n* = 9)	(*n* = 2)	(*n* = 2)	(*n* = 2)	(*n* = 2)	(*n* = 5)	(*n* = 5)

^a^All values of a category are calculated directly from the raw data. Therefore, the values cannot be calculated separately;

^b^n represents the number of considered trials.

The ADG of the non-vaccinated groups ranged from 438 to 970 g; the mean was 674 ± 163 g. In the vaccinated groups, the ADG ranged from 429 to 1,106 g, while the mean value was 700 ± 201 g, this corresponding to an increase of 3.9%. The feed conversion ratio of the animals in the non-vaccinated groups showed values between 1.87 and 3.81; the mean value was 2.70 ± 0.59. In comparison, the feed conversion ratio in the vaccinated groups ranged from 1.81 to 3.09, with a mean value of 2.58 ± 0.40, this corresponding to a decrease of 4.4%. The mortality rate in the non-vaccinated groups ranged from 2.4 to 7.5%; the mean was 4.3 ± 2.0%. In the vaccinated groups, the mortality rate ranged from 2.3 to 4.8%, while the mean value was 3.3 ± 1.1%, this corresponding to a reduction of 23.3%.

The calculation of the mean values of the carbon footprint resulted in a value of 3.36 kg CO_2_ eq per kg weight gain in the non-vaccinated groups and a value of 3.21 kg CO_2_ eq per kg weight gain in the vaccinated groups, the latter corresponding to a reduction of 4.5%.

#### 3.3.2 Publications concerning the rearing period

Data from two of the 14 trials could be included in the consideration concerning the rearing period. These were published in 2017 and 2018. The trials started at day 28 or 29 of life (mean: 29 days) and lasted 46 and 50 days (mean: 48 days), resulting in an age of 74 and 77 days of life (mean: 76 days) at the end of the trial (Table 4).

The ADG of the non-vaccinated groups in the rearing period ranged from 438 to 444 g with a mean value of 441 ± 3 g. In the vaccinated groups, the ADG ranged between 429 and 449 g, while the mean value was 439 ± 10 g, this corresponding to a reduction of 0.5%. The feed conversion ratio of the animals in the non-vaccinated group (*n* = 1) showed a value of 1.87. In comparison, the corresponding vaccinated group showed a value of 1.81, this corresponding to a reduction of 3.2%. The mortality rate of animals in the non-vaccinated groups showed values between 2.4 and 3.1%; the median and mean values were 2.8 ± 0.4%. In the epidemically infected groups with a vaccination, values between 2.7 and 4.8% were found, while the median and mean values were 3.8 ± 1.1%, this corresponding to an increase of 35.7%.

The calculation of the mean values of carbon footprint with the mean values resulted in a value of 1.90 kg CO_2_ eq per kg weight gain for the respective trial period in the non-vaccinated groups and a value of 1.87 kg CO_2_ eq per kg weight gain in the vaccinated groups, the latter corresponding to a reduction of 1.6%.

#### 3.3.3 Publications concerning the fattening period

Data from seven of the 14 trials, published from 2012 to 2020, could be included in the consideration concerning the fattening period. The trials started between day 67 and 75 of life and ended between day 143 and 194 of life, with a trial duration of 77–125 days. On average, the trials started at day 70 of life and ended at day 165 of life; the mean trial duration was 96 days (Table 4).

The ADG of non-vaccinated animals in the fattening period ranged from 858 to 970 g; the mean value was 914 ± 56 g. In the vaccinated groups, the ADG ranged between 922 and 1,106 g, while the mean value was 1,014 ± 92 g, this corresponding to an increase of 10.9%. The feed conversion ratio of the animals in the non-vaccinated groups showed values between 2.40 and 3.81; the mean value was 2.87 ± 0.50. In comparison, the equivalent vaccinated groups showed values between 2.37 and 2.93 and a mean value of 2.63 ± 0.22, this corresponding to a reduction of 8.4%. The mortality rate of animals in the non-vaccinated groups ranged from 3.1 to 7.5%; the mean value was 5.3 ± 2.2%. In the vaccinated groups, the mortality rate ranged between 2.3 and 2.6%, while the value was 2.5 ± 0.1%, this corresponding to a reduction of 52.8%.

The calculation of the mean values of the carbon footprint resulted in a value of 3.55 kg CO_2_ eq per kg weight gain for the respective trial period in the non-vaccinated groups and a value of 3.22 kg CO_2_ eq per kg weight gain in the vaccinated groups, the latter corresponding to a reduction of 9.3%.

#### 3.3.4 Publications concerning the rearing and fattening period

Data from five of the 14 trials, published from 2012 to 2022, could be included in the consideration concerning the rearing and fattening period. The trials started between day 21 and 28 of life and ended between day 161 and 186 of life, with a trial duration of 141–166 days. On average, the trials started at day 22 of life and ended at day 176 of life; the mean trial duration was 154 days (Table 4).

The ADG of the non-vaccinated groups in the rearing and fattening period was 612–728 g, while the mean was 671 ± 44 g. In the vaccinated groups, the ADG ranged between 616 and 732 g; the mean value was 678 ± 45 g, this corresponding to an increase of 1.0%. The mortality rate of animals in the non-vaccinated groups ranged from 2.4 to 6.7%; the mean value was 4.6 ± 1.9%. In vaccinated groups, the mortality rate ranged from 2.5 to 4.8%, while the mean value was 2.5 ± 0.1%, this corresponding to a reduction of 26.1%.

A calculation of the average values of the carbon footprint for these publications was not possible due to missing information on the feed conversion ratio.

### 3.4 Effects on the carbon footprint of a vaccination against *Lawsonia intracellularis*

#### 3.4.1 Consideration of all publications

In total, data from 17 trials, published from 2004 to 2022, fulfilled the criteria and were included in this evaluation regarding a vaccination against *LI* in epidemically infected situations (Table 1). The trials started between day 28 and 84 of life and ended between day 133 and 194 of life, with a trial duration of 56–166 days. On average, the trials started at day 72 of life and ended at day 178 of life; the mean trial duration was 106 days (Table 5).

**Table 5:** Means ± standard deviations of parameters (age of animals at start and end of trial, duration of trial, weight of animals at start and end of trial, ADG, feed conversion ratio, and mortality rate) in non-vaccinated and vaccinated groups of considered trials concerning an infection with *Lawsonia intracellularis* (ND = no data).

**Parameter^a^**	**Mean of all trials** ^**b**^		**Mean of trials for rearing period** ^**b**^		**Mean of trials for fattening period** ^**b**^		**Mean of trials for rearing and fattening period** ^**b**^	
Age at start of trial [days]	72 ± 18 (*n* = 14)		ND		79 ± 4 (*n* = 12)		29 ± 1 (*n* = 2)	
Age at end of trial [days]	178 ± 25 (*n* = 14)		ND		179 ± 26 (*n* = 12)		178 ± 17 (*n* = 2)	
Duration of trial [days]	106 ± 27 (*n* = 17)		ND		100 ± 23 (*n* = 15)		150 ± 16 (*n* = 2)	
Non-vaccinated (nv) or vaccinated groups (vac)	nv	vac	nv	vac	nv	vac	nv	vac
Body weight at start of trial [kg]	29.0 ± 9.7	29.1 ± 9.8	ND	ND	32.1 ± 5.3	32.3 ± 5.6	7.0 ± 0.0	7.1 ± 0.0
	(*n* = 17)	(*n* = 17)			(*n* = 15)	(*n* = 15)	(*n* = 2)	(*n* = 2)
Body weight at end of trial [kg]	116.0 ± 13.2	93.3 ± 14.1	ND	ND	117.3 ± 13.4	120.7 ± 14.3	106.3 ± 6.0	110.3 ± 6.3
	(*n* = 17)	(*n* = 17)			(*n* = 15)	(*n* = 15)	(*n* = 2)	(*n* = 2)
Average daily gain [g per day]	643 ± 136	119.4 ± 124	ND	ND	880 ± 142	902 ± 130	847 ± 61	878 ± 60
	(*n* = 17)	(*n* = 17)			(*n* = 15)	(*n* = 15)	(*n* = 2)	(*n* = 2)
Feed conversion ratio [kg per kg]	2.76 ± 0.30	2.69 ± 0.30	ND	ND	2.79 ± 0.30	2.71 ± 0.31	2.51 ± 0.14	2.47 ± 0.16
	(*n* = 17)	(*n* = 17)			(*n* = 15)	(*n* = 15)	(*n* = 2)	(*n* = 2)
Mortality rate [%]	3.6 ± 3.7	2.5 ± 2.5	ND	ND	3.8 ± 3.8	2.6 ± 2.5	1.2 ± 0.0	1.2 ± 0.0
	(*n* = 17)	(*n* = 17)			(*n* = 15)	(*n* = 15)	(*n* = 2)	(*n* = 2)

^a^All values of a category are calculated directly from the raw data. Therefore, the values cannot be calculated separately.

^b^n represents the number of considered trials.

The ADG of the non-vaccinated groups was between 643 and 1,106 g; the mean was 876 ± 136 g. In the groups that also received a vaccination against *LI*, the ADG values ranged between 657 and 1,109 g, while the mean value was 899 ± 124 g, this corresponding to an increase of 2.6%. The feed conversion ratio of the animals in the non-vaccinated groups showed values between 2.06 and 3.32; the mean value was 2.76 ± 0.30. In comparison, the corresponding vaccinated groups showed values from 1.96 to 3.23, while the mean value was 2.69 ± 0.30, this corresponding to a reduction of 2.5%. The mortality rate of the non-vaccinated groups ranged from 0.7 to 13.0%; the mean value was 3.6 ± 3.7%. In the vaccinated groups, the mortality rate ranged from 0.4 to 10.0%, while the mean value was 2.5 ± 2.5%, this corresponding to a reduction of 30.6%.

The calculation of the mean values of the carbon footprint resulted in a value of 3.44 kg CO_2_ eq per kg weight gain in the non-vaccinated groups and a value of 3.33 kg CO_2_ eq per kg weight gain in the vaccinated groups, the latter corresponding to a reduction of 3.2%.

#### 3.4.2 Publications concerning the rearing period

None of the considered studies relating to *LI* were exclusively concerned with the rearing period. Therefore, a corresponding analysis was not possible.

#### 3.4.3 Publications concerning the fattening period

Data from 15 of the 17 trials, published from 2012 to 2020, could be included in the consideration concerning the fattening period. The trials started between day 75 and 84 of life and ended between day 133 and 194 of life, with a trial duration of 56 to 120 days. On average, the trials started at day 79 of life and ended at day 179 of life; the mean trial duration was 100 days (Table 5).

The ADG of the pigs in the fattening period without a vaccination ranged from 643 g to 1106 g; the mean was 880 ± 142 g. In the vaccinated groups, the ADG ranged from 657 g to 1109 g, while the mean value was 902 ± 130 g, this corresponding to an increase of 2.5%. The feed conversion ratio of the animals in the non-vaccinated groups showed values between 2.06 and 3.32; the mean value was 2.79 ± 0.30. In comparison, the feed conversion rate in the vaccinated groups ranged from 1.96 to 3.23 with a mean value of 2.71 ± 0.31, this corresponding to a reduction of 2.9%. The mortality rate of animals without a vaccination ranged from 0.7 to 13.0%; the mean value was 3.8 ± 3.8%. In the vaccinated groups, the mortality rates ranged between 0.4 and 10.0%, while the mean was 2.6 ± 2.5%, this corresponding to a reduction of 31.6%.

The calculation of the carbon footprint with the mean values resulted in a value of 3.43 kg CO_2_ eq per kg weight gain for the respective trial period in the non-vaccinated groups and a value of 3.31 kg CO_2_ eq per kg weight gain in the vaccinated groups, the latter corresponding to a reduction of 3.5%.

#### 3.4.4 Publications concerning the rearing and fattening period

Data from two of the 17 trials could be included in the consideration concerning the rearing and fattening period. These were published in 2018 and 2022. The trials started at day 28 or 29 of life (mean: 29 days) and lasted 134 and 166 days (mean: 150 days), resulting in an age of 161 and 194 days of life (mean: 178 days) at the end of the trial (Table 5).

The ADG of the non-vaccinated groups in the rearing and fattening period (*n* = 2) was 785 and 908 g; the mean value was 847 ± 61 g. In the vaccinated groups, the ADG was 818 and 939 g, the mean value was 878 ± 60 g, this corresponding to an increase of 3.7%. The feed conversion ratio of the animals in the non-vaccinated groups showed values of 2.38 and 2.65; the mean value was 2.51 ± 0.14. In comparison, the feed conversion ratio in the corresponding vaccinated groups was 2.31 and 2.63, with a mean of 2.47 ± 0.16, this corresponding to a reduction of 1.6% (median/mean value). The mortality rate of animals amounted to 1.2% in both groups (vaccinated and non-vaccinated).

The calculation of the mean values of the carbon footprint resulted in a value of 3.33 kg CO_2_ eq per kg weight gain for the respective trial period in the non-vaccinated groups and a value of 3.28 kg CO_2_ eq per kg weight gain in the vaccinated groups, the latter corresponding to a reduction of 1.5%.

### 3.5 Estimations of the effects on the carbon footprint by vaccinations against infectious diseases of fattening pigs in Germany using scenario calculations

The annual savings potential was estimated using the above-mentioned percentage reductions concerning the fattening period or the rearing and the fattening period and a standard carbon footprint of 3.6 kg CO_2_ eq per kg weight gain. The reductions concerning the rearing period only were omitted from this consideration due to a limited amount of data.

A vaccination against PCV2 led to an average saving of 10 kg CO_2_ eq per 100 kg weight gain in the fattening period of the pigs and an average saving of 44 kg CO_2_ eq per 100 kg weight gain in the entire life cycle (Table 6). Correspondingly, this resulted in a theoretical annual savings potential of 327 thousand (k) metric tons CO_2_ eq (fattening period) or rather 1,465 k metric tons CO_2_ eq (entire life cycle) resulting from vaccinating pigs in Germany.

**Table 6:** Summary of effects of vaccination in epidemically infected situations on carbon footprint of fattening pigs under consideration of different lifetimes, including estimation of possible annual savings potential through a vaccination in Germany (ND, no data).

**Vaccination against infectious disease**	**Mean change of carbon footprint …**			**Scenario A (considering fattening period)** ^**a**^		**Scenario B (considering entire life cycle)** ^**a**^	
	**… in rearing period ^*a*^**	**… in fattening period ^*a*^**	**… in entire life cycle ^*a*^**	**Mean change in carbon footprint (per 100 kg weight gain)^*b*^**	**Theoretical annual savings potential through a vaccination in Germany^*c*^ (k = thousands)**	**Mean change in carbon footprint (per 100 kg weight gain)^b^**	**Theoretical annual savings potential through a vaccination in Germany^c^ (k = thousands)**
PCV2	−4.9%	−2.7%	−12.1%	−10 kg	−327 k metric tons	−44 kg	−1,465 k metric tons
				CO_2_ eq	CO_2_ eq	CO_2_ eq	CO_2_ eq
*M. hyo*	ND	−2.5%	ND	−9 kg	−303 k metric tons	ND	ND
				CO_2_ eq	CO_2_ eq		
PCV2 + *M. hyo*	−2.1%	−9.3%	ND	−33 kg	−1,126 k metric tons	ND	ND
				CO_2_ eq	CO_2_ eq		
*LI*	ND	−3.5%	−1.5%	−13 kg	−424 k metric tons	−5 kg	−182 k metric tons
				CO_2_ eq	CO_2_ eq	CO_2_ eq	CO_2_ eq

^a^Each publication was exclusively assigned to one category depending on age of pigs in trial period.

^b^Assumption: standard carbon footprint of 3.6 kg CO_2_ eq per kg weight gain (calculated using standard values described in Materials and methods).

^c^Assumptions: 43.8 million slaughtered fattening pigs in Germany (39) with a carcass weight of 97.2 kg und and a mean carcass yield of 79% (40).

From the publications on vaccination against *M. hyo*, a reduction of 9 kg CO_2_ eq per 100 kg weight gain may occur in the fattening period (no data for the entire lifetime). This led to a theoretical annual savings potential of 303 k metric tons CO_2_ eq during the fattening period as a result of vaccinating pigs in Germany.

A combination vaccination against PCV2 and *M. hyo* corresponded to a saving of 33 kg CO_2_ eq per 100 kg weight gain in the fattening period, this corresponding to a theoretical annual savings potential of 1,126 k CO_2_ eq during the fattening period resulting from vaccinating pigs in Germany. No data were available concerning long-term trials for the rearing and the fattening period.

A vaccination against *LI* resulted in a reduction of 13 kg CO_2_ eq per 100 kg weight gain in the fattening period and a reduction of 5 kg CO_2_ eq per 100 kg weight gain for the entire life cycle. This led to a theoretical annual savings potential of 424 k metric tons CO_2_ eq (fattening period) or 182 k metric tons CO_2_ eq (entire life cycle) as a result of vaccinating pigs in Germany.

## 4 Discussion

The growing world population will probably lead to a higher demand for food of animal origin in the coming years (41, 42). Due to its relatively high ecological footprint, livestock farming is one of the most controversial production chains for human food (43). Therefore, agriculture in general and livestock production in particular need to maximize the food security while minimizing the negative impact on the environment at the same time (43). This development also increases the need to align existing processes and potential growth even more clearly with efficiency standards. Maintaining animal health can be an important tool to ensure high efficiency. Infections may have a significant impact on performance due to reduced feed intake and inefficient nutrient utilization (7, 44). Thus, a reasonable vaccination program may be an important tool to not only maintain animal health but also secure a high standard of sustainability.

This evaluation deals with the effects of vaccination against PCV2, *M. hyo*, PCV2 and *M. hyo*, and *LI* in fattening pigs as an example for infectious diseases in general. The aim of the present literature study was to analyze the potential effects of the prevention of diseases on health and performance parameters and to identify concrete change in the environmental impact of fattening pigs under corresponding conditions. The evaluation used data from scientific publications on important pathogen-associated diseases causing severe issues and high economic losses in the rearing and fattening period of growing pigs. The publications and the results focused primarily on Europe. The evaluated diseases cover different organ systems and differ significantly in the course, duration, and severity of the disease.

In this evaluation, a vaccination against PCV2 led to a higher mean ADG in the rearing (+0.3%, *n* = 2) and the fattening period (+3.9%, *n* = 4). An increase in ADG following a vaccination was postulated from a review using publications between 2006 and 2008 (45) and a more recent meta-analysis with data from 2006 to 2014 (46). Both of those sources also indicated an interaction with co-infections with other common swine pathogens such as the porcine reproductive and respiratory disease syndrome virus (PRRSV), which was not included in our evaluation. In all of the studies under consideration, the ADG was higher in finishing pigs and in nursery-finishing pigs than during the rearing period as was found in our evaluation. The FCR in this evaluation was seen to decrease in both periods (rearing: −6.0%, *n* = 2; fattening −3.0%, *n* = 4). The mean mortality rate of the animals in this evaluation decreased by 22.6% in the rearing period (*n* = 1) and by 13.0% in the fattening period (*n* = 4), showing the same tendency as was postulated by the above mentioned review (45). Considering these three parameters, a vaccination against PCV2 resulted in pigs having a better overall performance with a higher efficiency and fewer losses in both phases. Former studies mainly used ADG and mortality rate to indicate the potential of vaccinations against PCV2; the FCR was not the main subject of consideration (45, 46). Concerning the carbon footprint, leaving out the FCR may result in a lack of information, as FCR is known to be a huge impact factor in former LCA studies on growing pigs (47–49). In this evaluation the changes in ADG, FCR, and mortality rate resulted in a reducing potential of the carbon footprint of around 5% regarding the rearing period of the pigs and of around 3% when considering the fattening period of the pigs. An even higher potential of around 12% could be derived from the publications dealing with the rearing and the fattening period in one trial. In those publications (*n* = 6) the mean ADG increased by 5.3% following a vaccination, while the FCR was reduced by 6.2% and the mortality rate showed a reduction of 57.3%. Therefore, the effect of transfer to a better performance was higher for those publications than for publications dealing with shorter time frames considering the rearing or fattening period only. As PCV2 is known to persist in pigs during their entire life and lead to an overall depression of the performance, a longer consideration of infected or rather vaccinated pigs may lead to greater effect on the carbon footprint (11, 15, 50). Focusing on the sustainability only, a vaccination as early as possible and permitted would lead to the highest reduction of the carbon footprint. In general, a prevention of PCV2 through a vaccination has the potential to increase the ADG, decrease the FCR, and decrease the mortality rate over a long period, resulting in a significant reduction of the carbon footprint (19, 45, 46, 50).

When considering the vaccination against *M. hyo*, for the fattening period data on all parameters were available. However, concerning ADG and mortality rate, data were only available from publications dealing with the rearing and the fattening period in one trial. The ADG increased following the vaccination (fattening: +3.0%, *n* = 3; rearing/fattening period: +2.3%, *n* = 6). A similar change was also postulated by a review from 2012 (51), In our evaluation, an improvement following the vaccination could also be noted concerning the mortality rate (fattening: −17.2%, *n* = 3; rearing/fattening: −9.4%, *n* = 3) and the FCR (fattening: −2.5%). Therefore, all performance parameters improved following the vaccination as is described in former reviews concerning that vaccination (29, 52). As *M. hyo* is known to cause high morbidity but low mortality rate compared to other diseases (8), the potential to improve this parameter is limited. The improvement in all three parameters resulted in a 2.5% lower carbon footprint using the mean values of the fattening period. As negative effects on the performance due to an infection with *M. hyo* may especially be found in the later stages of life (30), leaving out the rearing period in the consideration results in a comparably low loss of information.

A combined vaccination against PCV2 and *M. hyo* resulted in a mean ADG increase of 0.5% in the rearing period (*n* = 2), 3.0% in publications focusing on the fattening period only (*n* = 2), or 2.3% in the rearing and the fattening period (*n* = 4). The same pattern may be found concerning the mortality rate (rearing: +35.7%, *n* = 2; fattening: −52.8%, *n* = 2; rearing/fattening: −26.1%, *n* = 5). The FCR decreased in the rearing and the fattening period (rearing: −3.2%; *n* = 1; fattening: −8.4%, *n* = 5). Therefore, the combined vaccination indicated similar positive changes in ADG, FCR, and mortality as did single vaccinations against PCV2 or *M. hyo*, and might also show beneficial effects due to the reduction of treatments (53, 54). As the consideration of vaccinations against PCV2 and *M. hyo* as well as the combined vaccination against PCV2 and *M. hyo* in this evaluation are based on different publications from different countries and years, a comparison between the effects on ADG, FCR, and the mortality rate between different vaccination types is inappropriate. The carbon footprint decreased by 2.1% following the vaccination in the rearing period and by 9.3% in the fattening period. Based on these results, it may be concluded that higher CO_2_ reduction potential may be found in the fattening period, which may be traced back to the relatively high changes in all three parameters. This applies to vaccinations with *M. hyo*, which have a greater impact in the fattening period (30). FCR is known to have a great impact on the carbon footprint of pigs (47–49). However, in this evaluation, unfortunately no carbon footprint could be calculated concerning long-term trials during the rearing and fattening period due to missing FCR values. In line with the changes in ADG and mortality rate, it may be assumed that the carbon footprint would decrease following a vaccination in those trials but by a lower percentage than in the fattening period.

No data could be considered regarding a vaccination against *LI* for the rearing period only. As infections with *LI* are more likely to be found in the later stages of life (33), omitting the rearing period from our evaluation results in a comparably low loss of information. The ADG increased in trials concerning the fattening period and trials focusing on the rearing and the fattening period (fattening: +2.5%, *n* = 15, rearing/fattening: +3.7%, *n* = 2), while the FCR decreased in both periods (fattening: −2.9%, *n* = 15, rearing/fattening: −1.6%, *n* = 2). Therefore, both parameters improved following a vaccination against *LI*. The same effects were described by a review article from 2005 (55). For the fattening period, this tendency was confirmed by the mortality rate showing a reduction of 31.6% (*n* = 15) according to a recent review article conducted on ADG and mortality rate (18). That review also confirmed a higher ADG following a vaccination against *LI*. In the trials performed during the rearing and the fattening period, no difference in mortality rate could be found regarding non-vaccinated and vaccinated groups (*n* = 2). The carbon footprint decreased by 3.5% (fattening period) and 1.5% (rearing/fattening period). That difference between the consideration of the fattening period or the rearing and the fattening period may be traced back to the relatively high differences concerning the mortality rate also driven by a greater reduction in FCR in trials concerning the fattening period. The ADG, however, would indicate a higher potential for the trials concerning the rearing and fattening period. This indicates that changes in the FCR have a greater impact on the carbon footprint than those in ADG and mortality rate as was suggested by former LCA studies on growing pigs (47–49).

The results regarding the vaccination against *LI* are a good example that the number of studies available can vary considerably, which must be taken into account when interpreting the results. Additionally, one must keep in mind that all effects were extracted from a variety of publications with regard to trial periods, breeds, or breeding lines used and countries of origin, which reduce the comparability or coherence of the trials. Therefore, the results of this analysis should be understood as an estimate of the effects only. Furthermore, there are of course some physiological limits (e.g., the feed conversion ratio cannot be reduced indefinitely), which rule out the transfer of the results to every context and an additivity of the effects.

The annual savings potential in Germany concerning all diseases differed from 182 k metric tons CO_2_ to 1,465 k metric tons CO_2_ eq. The small amount and wide spread of underlying data concerning time, age, country of origin, and housing conditions clearly limits the informative value. Furthermore, there are of course sometimes physiological limits (e.g., the amount of feed cannot be reduced indefinitely), which rule out the transfer of the results to any context and also an additivity of the effects. Therefore, assumptions were made when calculating the data and those values should be considered as rough estimates only. German agriculture was responsible for the emission of about 53.3 million metric tons CO_2_ eq in 2023 (56). Therefore, based on the rough estimate, vaccinating all fattening pigs has a theoretical potential to decrease this level by 0.3–2.7% (compared to non-vaccinated fattening pigs) and may thereby help to improve the ecological sustainability of fattening pigs.

Overall, the literature search and calculations in this review revealed a rather incomplete data situation. The majority of the studies had insufficient data depth, which means that many of them could not be included at all, resulting in a small number of included publications. Such a small number of publications of course decreases the available number of publications regarding, for example, the year of the trial or the country and therefore may not sufficiently illustrate the overall situation. The assumption concerning weight development and mortality, used to fulfill the data, and the assumed feed rations in the calculations may result in a possible distortion of the results. An attempt was made to minimize this by making assumptions that were as realistic as possible based on literature. Nevertheless, it is known from previous studies that the feed and the raw components used have a significant influence on carbon footprint. Therefore, the use of a different feed rations (e.g., taking into account raw materials with a comparable low carbon footprint) would significantly influence the level of the calculated carbon footprints, but would result in percentage changes of a similar magnitude. More recent and more detailed data concerning the performance of the pigs are needed to describe the effects of vaccinations against PCV2, *M. hyo*, and *LI* on the carbon footprint of fattening pigs more accurately and minimize the distortions caused by the assumptions. Nevertheless, this evaluation clearly shows that healthy animals can achieve a reduced carbon footprint through better performance with lower resource consumption and are therefore extremely important for sustainable animal husbandry. In order to reduce the carbon footprint of animal husbandry the health status of the animals should be focused on more. Good management of their farms and a high level of hygiene are therefore essential for farmers. The use of vaccinations can also be a useful and important tool for achieving this goal and must therefore be used in animal husbandry according to a sensible plan based on scientific findings in order to unsure healthy animals.

## Data Availability

The original contributions presented in the study are included in the article/Supplementary material, further inquiries can be directed to the corresponding author.
