# Supplementary material for: Influence of vaccination against infectious diseases on the carbon footprint of fattening pigs: a systematic review
Source: Front Vet Sci. 2024 Dec 18;11:1487742. doi: 10.3389/fvets.2024.1487742 (PMC11688352; doi:10.3389/fvets.2024.1487742)
Supplement: Supplementary file 1 [file Table_1.docx]

# Included publications (see also Table 1)

## Included publications on PCV2

Astrup P, Nielsen GB, Haugegaard J (2017). Porcilis® PCV improves Feed Conversion Rate in weaners. 9th European Symposium of porcine health management, May 3rd - 5th 2017, Prague, Czech Republic

Brons N, Neto R, Vila T, Longo S, Joisel F (2010). Improved production parameters and profit as a result of PCV2 piglet vaccination in the United Kingdom. 21st International pig veterinary society (IPVS) congress, July 18th – 21st 2010, Vancouver, Canada

Coll T, Martos A, Hernández-Caravaca I (2012). Effect of PCV2 piglet vaccination on FCR in 24 Spanish farms. 4th European Symposium of porcine health management, April 25th – 27th 2012, Bruges, Belgium

de Groot R, Wertenbroek N (2014). Reduction in PDNS cases after PCV2 vaccination on a Dutch fattening farm. 23rd International pig veterinary society (IPVS) congress, June 8th – 11st 2014, Cancun, Mexico

Fachinger V, Bischoff R, Jedidia SB, Saalmüller A, Elbers K (2007). The effect of vaccination against porcine circovirus type 2 in pigs suffering from porcine respiratory disease complex. Vaccine. 2008 Mar 10;26(11):1488-99. doi: 10.1016/j.vaccine.2007.11.053. Epub 2007 Dec 7. PMID: 18304705.

Fiebig K, Ernst G, Mischok J (2018). Impact of an intradermal PCV2 vaccine on various production parameters and antibiotic consumption in finishers. 10th European Symposium of porcine health management, May 9th – 11th 2018, Barcelona, Spain

Jansen R, Steenaert M (2020). Intervention with Ingelvac CircoFLEX® in a Dutch closed herd improved finishing pig performance. 26st International pig veterinary society (IPVS) congress, July 18th – 21st 2010, Rio de Janiero, Brasil

Kaalberg L, Geurts V, Jolie R (2017). A field efficacy and safety trial in the Netherlands in pigs vaccinated at 3 weeks of age with a ready-to-use porcine circovirus type 2 and Mycoplasma hyopneumoniae combined vaccine. Porcine Health Manag. 2017 Nov 9;3:23. doi: 10.1186/s40813-017-0070-5. PMID: 29152324; PMCID: PMC5679184.

Koenders K, Wertenbroek N (2012). Implementing PCV2 vaccination results in reduction of antibiotic use and improved technical results on a Dutch farrow-to-finish farm. 4th European Symposium of porcine health management, April 25th – 27th 2012, Bruges, Belgium

Lewandowski E, Jagu R, Adam M (2012). PCV2 piglet vaccination allows French swine herds to reduce their overall veterinary expenses. 4th European Symposium of porcine health management, April 25th – 27th 2012, Bruges, Belgium

Nielsen GB, Nielsen JP, Haugegaard J, Denwood MJ, Houe H (2017). Effect of vaccination against sub-clinical Porcine Circovirus type 2 infection in a high-health finishing pig herd: A randomised clinical field trial. Prev Vet Med. 2017 Jun 1;141:14-21. doi: 10.1016/j.prevetmed.2017.04.003. Epub 2017 Apr 15. PMID: 28532989.

Rahm A, Lamminger A, Palzer A, Ritzmann M, Fiebig K (2018). Significant reduction of PCV2-viraemia and improvement of production parameters during fattening following vaccination with a ready-use-PCV M Hyo vaccine. 10th European Symposium of porcine health management, May 9th – 11th 2018, Barcelona, Spain

Yao L, Dai H, Fang B, Huang N, Yang H (2010). Field experience with a subunit PCV2 vaccine in a Chinese farm with severe PCVD. 21st International pig veterinary society (IPVS) congress, July 18th – 21st 2010, Vancouver, Canada

## Included publications on *M. hyo*

Arsenakis I, Michiels A, Del Pozo Sacristán R, Boyen F, Haesebrouck F, Maes D (2017). Mycoplasma hyopneumoniae vaccination at or shortly before weaning under field conditions: a randomised efficacy trial. Vet Rec. 2017 Jul 1;181(1):19. doi: 10.1136/vr.104075. Epub 2017 Jun 10. PMID: 28601840.

Beek J, Claeyé E, Del Pozo R, Van Gorp S, Segers H (2017). Technical Performance Comparison in Finishers vaccinated with Porcilis® PCV M Hyo or other Vaccination Programs in 15 Belgian Pig Herds. 9th European Symposium of porcine health management, May 3rd - 5th 2017, Prague, Czech Republic

Beffort L, Weiß C, Fiebig K, Jolie R, Ritzmann M, Eddicks M (2017). Field study on the safety and efficacy of intradermal versus intramuscular vaccination against Mycoplasma hyopneumoniae. Vet Rec. 2017 Sep 30;181(13):348. doi: 10.1136/vr.104466. Epub 2017 Sep 11. PMID: 28893974

Herrera J, Figueras Gourgues S, Rodriguez-Vega V, Hernandez Caravaca I (2014). Large scale field observation of the efficacy of MycoFLEX® in an integrator company. 23rd International pig veterinary society (IPVS) congress, June 8th – 11st 2014, Cancun, Mexico

Kristensen CS, Vinther J, Svensmark B, Bækbo P (2014). A field evaluation of two vaccines against Mycoplasma hyopneumoniae infection in pigs. Acta Vet Scand. 2014 Apr 16;56(1):24. doi: 10.1186/1751-0147-56-24. PMID: 24739629; PMCID: PMC4012783.

Maes D, Deluyker H, Verdonck M, Castryck F, Miry C, Vrijens B, Verbeke W, Viaene J, de Kruif A (1999). Effect of vaccination against Mycoplasma hyopneumoniae in pig herds with an allin/all-out production system. Vaccine. 1999 Mar 5;17(9-10):1024-34. doi: 10.1016/s0264- 410x(98)00254-0. PMID: 10195611.

Potter ML, Kane EM, Bergstrom JR, Dritz SS, Tokach MD, Derouchey JM, Goodband RD, Nelssen JL (2012). Effects of diet source and vaccination for porcine circovirustype 2 and Mycoplasma hyopneumoniae on nursery pig performance. J Anim Sci. 2012 Nov;90(11):4063-71. doi: 10.2527/jas.2011-3943. Epub 2012 Jun 4. PMID: 22665636.

Struik D, Steenaert M, Wertenbroek N (2014). Additional Mycoplasma vaccination improves Dutch pig herd performance. 23rd International pig veterinary society (IPVS) congress, June 8th – 11st 2014, Cancun, Mexico

Tassis PD, Papatsiros VG, Nell T, Maes D, Alexopoulos C, Kyriakis SC, Tzika ED (2012). Clinical evaluation of intradermal vaccination against porcine enzootic pneumonia (Mycoplasma hyopneumoniae). Vet Rec. 2012 Mar 10;170(10):261. doi: 10.1136/vr.100239. Epub 2012 Jan 18. PMID: 22262700.

Tzika ED, Tassis PD, Koulialis D, Papatsiros VG, Nell T, Brellou G, Tsakmakidis I (2015). Field efficacy study of a novel ready-to-use vaccine against mycoplasma hyopneumoniae and porcine circovirus type 2 in a Greek farm. Porcine Health Manag. 2015 Nov 1;1:15. doi: 10.1186/s40813-015-0006-x. PMID: 28405421; PMCID: PMC5382375

## Included publications on PCV2 and *M. hyo*

Agerley M, Havn K, Rathkjen PH, Bak H (2012). Vaccination against PCV2 cost effectively reduces feed costs. 22nd International pig veterinary society (IPVS) congress, June 10th – 13th 2012, Jeju, Korea

Beek J, Claeyé E, Del Pozo R, Van Gorp S, Segers H (2017). Technical Performance Comparison in Finishers vaccinated with Porcilis® PCV M Hyo or other Vaccination Programs in 15 Belgian Pig Herds. 9th European Symposium of porcine health management, May 3rd - 5th 2017 Prague, Czech Republic

Boulbria G, Brilland S, Teixeira-Costa C, Brissonnier M, Charles M, Capdevielle N, Normand V, Bouchet F, Berton P, Krejci R, Lebret A (2021). Effectiveness of two intramuscular combined vaccines for the control of Mycoplasma hyopneumoniae and porcine circovirus type 2 in growing pigs: a randomized field trial. Porcine Health Manag. 2021 Jun 27;7(1):41. doi: 10.1186/s40813-021-00220-3. PMID: 34176520; PMCID: PMC8237417.

Cho H, Oh T, Suh J, Chae C (2022). A Comparative Field Evaluation of the Effect of Growth Performance Between Porcine Circovirus Type 2a (PCV2a)- and PCV2b-Based Bivalent Vaccines Containing PCV2 and Mycoplasma hyopneumoniae. Front Vet Sci. 2022 Jun 24;9:859344. doi: 10.3389/fvets.2022.859344. PMID: 35812885; PMCID: PMC9263624.

Duivon D., Pagot E., Trotel A., Rigaut M., Roudaut D., Eon L., Jolie R (2016). Efficacy of a new PCV2 and Mycoplasma hyopneumoniae combination vaccine: comparative fi eld study versus other common vaccines. 24th IPVS Congress, June 7th - 10th 2016, Dublin, Ireland

Ju JU, Kwak SH, Oh YS, Cho BJ, Bok VK (2012). Long term evaluations of circovirus and Mycolasma hyopneumonia vaccination program in Korean swine farm. 22nd International pig veterinary society (IPVS) congress, June 10th – 13th 2012, Jeju, Korea

Nielsen GB., Haugegaard J., Jolie R (2018). Field evaluation of a ready-to-use combined Porcine circovirus type 2 and Mycoplasma hyopneumoniae vaccine in Denmark – a historical comparison of productivity parameters in 20 nursery and 23 finishing herds. Porcine Health Manag. 2018; 4: 29. Published online 2018 Dec 7. doi: 10.1186/s40813-018-0104-7"

Pagot E, Rigaut M, Roudaut D, Panzavolta L, Jolie R, Duivon D (2017). Field efficacy of Porcilis® PCV M Hyo versus a licensed commercially available vaccine and placebo in the prevention of PRDC in pigs on a French farm: a randomized controlled trial. Porcine Health Manag. 2017 Feb 1;3:3. doi: 10.1186/s40813-016-0051-0. PMID: 28405459; PMCID: PMC5382521.

Pelz et al. (2020). Auswertung von Produktionsdaten im PRDC Problembetrieb nach Umstellung der PCV2 Mycoplasma hyopneumoniae Prophylaxe auf Porcilis® PCV M Hyo Variodose; Tierärztliche Umschau Pferd und Nutztier 4 2020, 28-32

Tassis PD, Tsakmakidis I, Papatsiros VG, Koulialis D, Nell T, Brellou G, Tzika ED (2017). A randomized controlled study on the efficacy of a novel combination vaccine against enzootic pneumonia (Mycoplasma hyopneumoniae) and porcine Circovirus type 2 (PCV2) in the presence of strong maternally derived PCV2 immunity in pigs. BMC Vet Res. 2017 Apr 7;13(1):91. doi: 10.1186/s12917-017-1014-7. PMID: 28388953; PMCID: PMC5384188.

Van Hee E., Woolfenden N., Berkshire D., Evans R., Neto R., Jolie, R (2016). Comparative efficacy of a ready to use and freshly mixed PCV2 and Mycoplasma hyopneumoniae vaccine. 24th IPVS Congress, June 7th - 10th 2016, Dublin, Ireland

## Included publications on *LI*

Antonelli A, Bianchi M, Leotti G, Andreoni S (2021). Impact of Oral Vaccination with Enterisol Ileitis on Growth Parameters and Antibiotic Consumption in Italian Fattening Farms. 12th European Symposium of porcine health management, April 14th – 16th 2021, online

Gómez JD, Lopéz JC, Sanchez ME, Giraldo JF, Naranja R JF, Collell M, Montoya M (2010). Effect of vaccination with Porcilis Ileitis on productive parameters in fattening in a commercial farm. 26st International pig veterinary society (IPVS) congress, July 18th – 21st 2010, Rio de Janiero, Brasil

Kolb (2004). Proliferative Enteropathy (Ileitis) in Finisher Pigs: An Evaluation of Vaccination in Live Production in the USA. 18th International pig veterinary society (IPVS) congress, June 27th – July 1st 2004, Hamburg, Germany

Marcos M, Jiménez M, Menjón R, Llorente C, Pérez ML, Santos L, Serrano D, Tejedor T (2022). Control of ileitis in growers and finisher pigs after Lawsonia intracellularis parenteral vaccination. 14th European Symposium of porcine health management, May 11th - 13th 2022 Thessaloniki, Greece

Meschede J, Holtrup S, Deitmer R, Mesu AP, Kraft C (2021). Reduction of Salmonella prevalence at slaughter in Lawsonia intracellularis co-infected swine herds by Enterisol® Ileitis vaccination. Heliyon. 2021 Apr 14;7(4):e06714. doi: 10.1016/j.heliyon.2021.e06714. PMID: 33912707; PMCID: PMC8066352.

Musse SL, Nielsen GB, Stege H, Weber NR, Houe H (2023). Effect of intramuscular vaccination against Lawsonia intracellularis on production parameters, diarrhea occurrence, antimicrobial treatment, bacterial shedding, and lean meat percentage in two Danish naturally infected finisher pig herds. Prev Vet Med. 2023 Mar;212:105837. doi: 10.1016/j.prevetmed.2023.105837. Epub 2023 Jan 6. PMID: 36680993.

Musse SL, Nielsen GB, Stege H, Weber NR, Houe H (2023). Productivity parameters, antimicrobial consumption, and prevalence of enteric pathogens before and after intramuscular vaccination against Lawsonia intracellularis in naturally infected Danish weaner and finisher pig herds. Preventive Veterinary Medicine 217 (2023) 105973

Nieberding (2022). Praktische Beobachtungen zum Einsatz einer intradermalen Lawsonia Impfung. Tierärztliche Umschau Pferd und Nutztier 3 2022

Peiponen KS, Tirkkonen BT, Junnila JJT, Heinonen ML (2018). Effect of a live attenuated vaccine against Lawsonia intracellularis in weaned and finishing pig settings in Finland. Acta Vet Scand. 2018 Mar 23;60(1):18. doi: 10.1186/s13028-018-0374-8. PMID: 29566718; PMCID: PMC5865362.

Raymakers & Kraneburg (2016). Field observation: Successful oral ileitis vaccination in liquid feed. 24th International pig veterinary society (IPVS) congress, June 7th – 10th 2016, Dublin, Ireland
