# Supplementary material for: Influence of vaccination against infectious diseases on the carbon footprint of fattening pigs: a systematic review
Source: Front Vet Sci. 2024 Dec 18;11:1487742. doi: 10.3389/fvets.2024.1487742 (PMC11688352; doi:10.3389/fvets.2024.1487742)
Supplement: Supplementary file 3 [file Table_3.docx]

**Identification of studies via databases and registers (PCV2)**

Records removed *before screening*:

Duplicate records removed
(n = 1)

Records identified from:

PubMed (n = 29)

**Identification**

Records screened

(n = 28)

Records excluded

Review Articles (n = 2)

Reports sought for retrieval

(n = 26)

Reports not retrieved

Inappropriate trial design (n = 6)

**Screening**

Reports excluded:

No vaccination (n = 6)

Reports assessed for eligibility

(n = 20)

Reports of included studies

(n = 14)

**Included**

Source: Page MJ, et al. BMJ 2021;372:n71. doi: 10.1136/bmj.n71.

This work is licensed under CC BY 4.0. To view a copy of this license, visit <https://creativecommons.org/licenses/by/4.0/>

**Identification of studies via databases and registers (*M. hyo*)**

Records removed *before screening*:

Duplicate records removed
(n = 0)

Records identified from:

PubMed (n = 23)

**Identification**

Records screened

(n = 23)

Records excluded

Published before 1990 (n = 1)

Reports sought for retrieval

(n = 22)

Reports not retrieved

Inappropriate trial design

(n = 4)

**Screening**

Reports excluded:

No vaccination (n = 2)

No performance data (n=2)

Reports assessed for eligibility

(n = 18)

Reports of included studies

(n = 14)

**Included**

Source: Page MJ, et al. BMJ 2021;372:n71. doi: 10.1136/bmj.n71.

This work is licensed under CC BY 4.0. To view a copy of this license, visit <https://creativecommons.org/licenses/by/4.0/>

**Identification of studies via databases and registers (PCV2 and *M. hyo*)**

Records removed *before screening*:

Duplicate records removed
(n = 1)

Records identified from:

PubMed (n = 25)

**Identification**

Records screened

(n = 24)

Records excluded

Review Articles (n = 0)

Reports sought for retrieval

(n = 24)

Reports not retrieved

Inappropriate trial design (n = 9)

**Screening**

Reports excluded:

No vaccination (n = 1)

Reports assessed for eligibility

(n = 15)

Reports of included studies

(n = 14)

**Included**

Source: Page MJ, et al. BMJ 2021;372:n71. doi: 10.1136/bmj.n71.

This work is licensed under CC BY 4.0. To view a copy of this license, visit <https://creativecommons.org/licenses/by/4.0/>

**Identification of studies via databases and registers (*LI*)**

Records removed *before screening*:

Duplicate records removed
(n = 2)

Records identified from:

PubMed (n = 35)

**Identification**

Records screened

(n = 33)

Records excluded

Review Articles (n = 0)

Reports sought for retrieval

(n = 33)

Reports not retrieved

Inappropriate trial design

(n = 11)

**Screening**

Reports excluded:

No vaccination (n = 3)

No performance data (n=2)

Reports assessed for eligibility

(n = 22)

Reports of included studies

(n = 17)

**Included**

Source: Page MJ, et al. BMJ 2021;372:n71. doi: 10.1136/bmj.n71.

This work is licensed under CC BY 4.0. To view a copy of this license, visit <https://creativecommons.org/licenses/by/4.0/>
